# Efficacy of acupuncture in the treatment of asthenospermia in obese men: study protocol for a randomized control trial

**DOI:** 10.3389/fmed.2026.1749806

**Published:** 2026-03-11

**Authors:** Hao Wang, Yongqing Zhao, Hongyuan Chang, Wenguang Zhou, Jun Guo, Linda Zhong

**Affiliations:** 1 Guo Jun Qihuang Scholar Inheritance Studio, Ningxia Hui Autonomous Region Hospital of Traditional Chinese Medicine, Ningxia Hui Autonomous Region Academy of Traditional Chinese Medicine, Ningxia, China; 2 Department of Andrology, Xiyuan Hospital of China Academy of Chinese Medical Sciences, Beijing, China; 3 Graduate School of Beijing University of Chinese Medicine, Beijing, China; 4 Biomedical Sciences and Chinese Medicine, School of Biological Sciences, Nanyang Technological University, Singapore, Singapore

**Keywords:** acupoint, acupuncture, asthenospermia, male infertility, obesity

## Abstract

**Background:**

Asthenospermia is one of the most common causes of male infertility. In recent years, with changes in dietary habits, the number of obese patients with asthenospermia has been increasing, leading to a decline in male fertility. However, there remains a lack of safe and effective treatments for this condition.

**Objective:**

This study aims to confirm the efficacy and safety of acupuncture in obese patients with asthenospermia.

**Methods:**

In our randomized controlled trial, 72 patients will be randomly assigned (1:1) to receive either acupuncture treatment or sham acupuncture treatment. Each group will receive treatment two times weekly for twelve weeks with another twelve weeks for follow-up. The primary outcome is the progressive sperm motility (PR). And the secondary outcomes include PR plus non-progressive sperm motility, sperm concentration, semen volume, sperm morphology, body mass index, waist-to-hip ratio, and body fat percentage. We will also evaluate adverse events that occur during the acupuncture process.

**Results:**

This study is expected to demonstrate whether acupuncture is effective and safe in the treatment of asthenospermia in obese men.

**Conclusion:**

The research findings will firstly provide new therapeutic evidence in treating asthenospermia of obese men and offer an alternative treatment options for improving the fertility of obese men.

**Study protocol registration:**

https://itmctr.ccebtcm.org.cn, identifier ITMCTR2025002025.

## 1 Introduction

Obesity is a critical global health issue affecting over one billion people (1). It is linked to a lot of comorbidities impacting every organ system and doubles annual medical costs compared to individuals with normal weight in adults (2, 3). The global rise in obesity stems from interconnected factors, primarily characterized by high-calorie junk food with excess fat and sugars, lack of enough physical activity and other unhealthy lifestyles such as sleep deprivation, smoking, and excessive drinking (4). In recent years, the impact of obesity on male reproductive health has been extensively studied. Clinical evidence indicated a dose-response relationship between increasing body mass index (BMI) and subfertility (5, 6). Hammiche et al. (7) reported that overweight was negatively associated with the percentage of progressive motility type A (*P* = 0.036) and positively associated with the percentage of immotility type C (*P* = 0.002). Ramaraju et al. (8) reviewed computer-aided sperm analysis data from 1,285 men and found that obesity was associated with lower progressive motility and total motility. Additionally, obese men were more likely to have asthenospermia and oligospermia (8). The mechanism by which obesity affects male semen quality may be related to aggravated oxidative stress, sperm DNA damage, decreased mitochondrial activity, and epigenetic changes (9).

The treatment of asthenospermia in obese men includes lifestyle interventions, medication, and weight loss surgery (10). Physical exercise may be generally associated with improved markers of male fertility, though outcomes may depend on the form and intensity of activity (11). Metformin and GLP-1 agonists exhibit ambiguous impacts, with evidence pointing to both positive and negative influences on reproductive health in men (12). Also, surgical approaches such as bariatric surgery are beneficial for correcting hormonal imbalances, yet their effects on semen parameters remain inconsistent (13). Therefore, identifying effective interventions that concurrently mitigate obesity and improve semen quality remains a central challenge in enhancing male fertility. Acupuncture has been widely used in some Asian countries. In previous reviews, we have analyzed the role of acupuncture in andrological diseases, and its mechanism included regulating sex hormone levels, modulating the autonomic nervous system, and improving oxidative stress (14, 15). Acupuncture can also improve the semen quality through enhancing the function of cation channels in spermatozoa, then leading to increased calcium ion influx and improved sperm motility (16). Although some studies have shown that acupuncture can improve sperm motility and body mass index (BMI) in men (17, 18), its effect on semen quality in obese men remains unknown. Therefore, we designed this randomized controlled trial to evaluate the impact of acupuncture on semen quality in obese men with asthenospermia.

## 2 Materials and methods

### 2.1 Study design and settings

**FIGURE 1:**
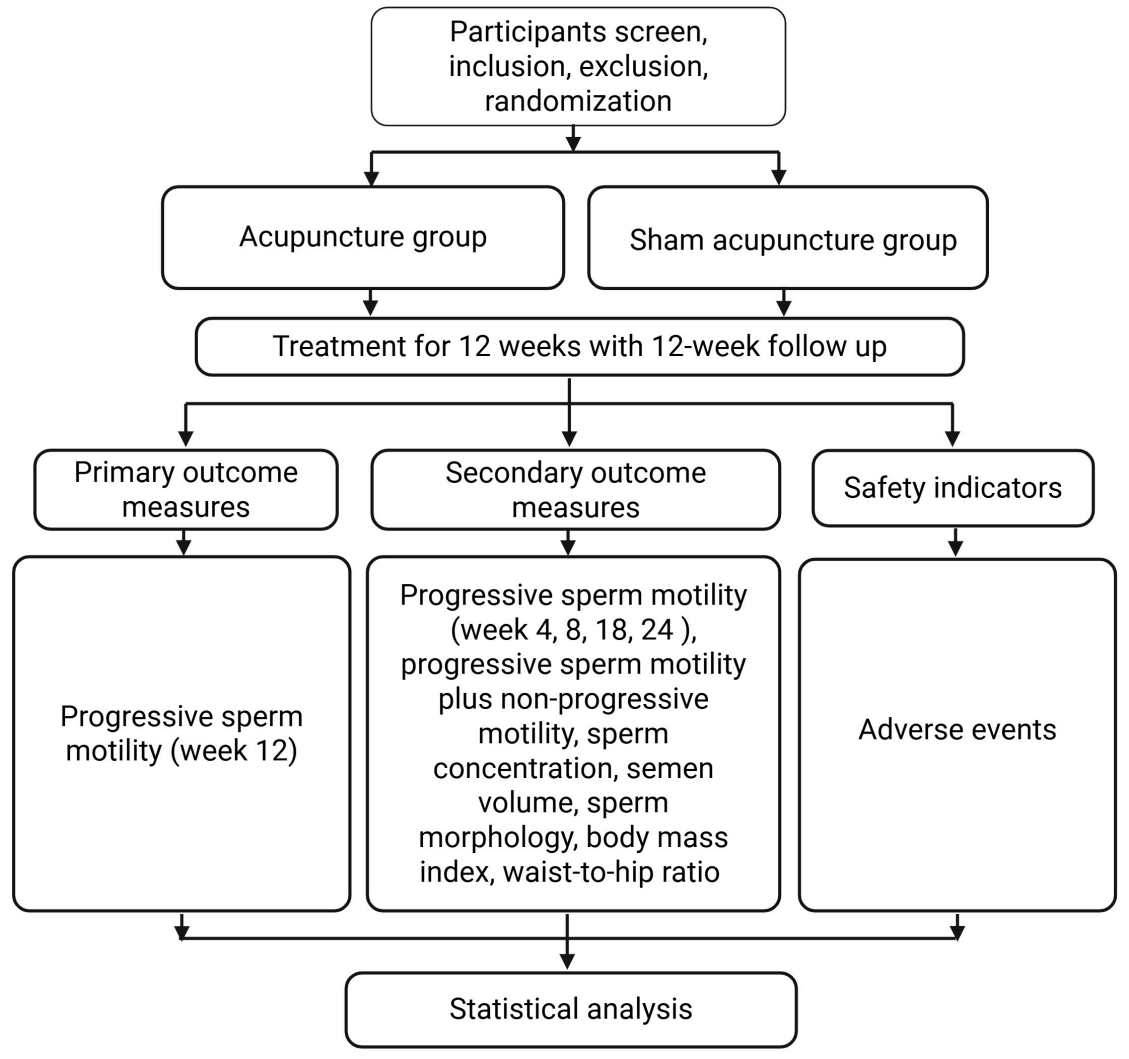
Study flowchart.

Our study is a 1:1 randomized controlled trial with two parallel groups and will be reported in accordance with the Standards for Reporting Interventions in Clinical Trials of Acupuncture and the SPIRIT 2013 Statement (19, 20). A total of 72 participants will be recruited for this study, with 36 participants assigned to each group. All participants will receive a course of acupuncture treatment after being informed about the study, risks, and benefits, and after signing an informed consent form. The trial flow chart and time point of the assessment are shown in Figure 1 and Table 1.

**TABLE 1:** A process chart of the trial.

Timepoint	Enrollment	Allocation	Treatment phase			Follow-up phase	
	Week-1	Week 0	Week 4	Week 8	Week 12	Week 18	Week 24
**Enrollment**							
Inclusion/exclusion criteria	X						
Informed consent	X						
Physical examination	X						
Randomization and allocation	X						
Demographic characteristics	X						
Allocation		X					
**Intervention**							
Acupuncture			X	X	X	X	X
Sham acupuncture			X	X	X	X	X
**Assessment**							
PR		X	X	X	X	X	X
PR plus NP		X	X	X	X	X	X
Sperm concentration		X	X	X	X	X	X
Semen volume		X	X	X	X	X	X
Sperm morphology		X	X	X	X	X	X
BMI		X	X	X	X	X	X
waist-to-hip ratio		X	X	X	X	X	X
Body fat percentage		X	X	X	X	X	X
Adverse events			X	X	X	X	X

BMI, body mass index; PR, progressive sperm motility; NP, non-progressive sperm motility.

### 2.2 Study participants

#### 2.2.1 Recruitment strategy

We will screen and recruit participants from November 2025 to October 2026 at the Department of Andrology, Xiyuan Hospital, China Academy of Chinese Medicine Sciences. Our researchers will be systematically trained and fully informed of the trial procedures. Informed consent will be obtained from participants prior to enrollment. Participation will be entirely voluntary, and participants will have the right to withdraw from the trial at any time without penalty.

#### 2.2.2 Diagnostic criteria

Diagnostic criteria for asthenospermia and obesity were established in accordance with the World Health Organization Manual for the Examination and Processing of Human Semen (5th Edition) and the “Health Industry Standard of the People’s Republic of China–Measurement of Adult Body Weight” (Standard No. WS/T428-2013) (21, 22). All participants must meet both the diagnostic criteria for asthenospermia and obesity simultaneously.

Diagnostic criteria for asthenospermia: Based on two or more semen analyses, the percentage of progressive motility spermatozoa in the semen is below the reference lower limit of 32%.

Diagnostic criteria for obesity: Based on body mass index (BMI = weight/height^2^), the BMI ≥ 28 kg/m^2^ is defined as obesity.

#### 2.2.3 Inclusion criteria

The study inclusion criteria are as follows: (1) Meanwhile meet the diagnostic criteria for both asthenospermia and obesity; (2) 22 years old ≤ age ≤ 45 years old; (3) No fertility needs in the past six months; (4) Voluntarily sign the informed consent form.

#### 2.2.4 Exclusion criteria

This study exclusion criteria are as follows: (1) Congenital chromosomal abnormalities or microdeletions of the Y chromosome or other known hereditary diseases that may affect semen quality; (2) Combined with oligospermia or obstructive azoospermia; (3) Combined with moderate to severe varicocele cryptorchidism or orchitis or epididymitis or mycoplasma or chlamydia or other diseases that clearly affect semen quality; (4) Combined with diabetes mellitus requiring long-term medication to control blood glucose; (5) Combined with serious cardiovascular diseases or serious cerebrovascular diseases or hematopoietic system diseases or psychiatric diseases or other complications; (6) Receipt of other relevant treatments for this disease such as aromatase inhibitors or estrogen antagonists etc. two weeks prior to treatment; (7) Fear of needles or fainting during acupuncture treatment in the past.

#### 2.2.5 Termination criteria

The termination criteria are as follows: (1) Participants voluntary withdrawal, such as revoking informed consent; (2) Participants whose condition progressively worsens during the trial, and for whom the physician determines discontinuation of the clinical trial is necessary. To protect the subject, they should withdraw from the trial and receive alternative treatment; (3) Participants develop certain comorbidities, complications, or specific physiological changes rendering continued participation inappropriate during the trial; (4) During the trial, participants demonstrate poor compliance, fails to undergo acupuncture intervention as specified, or uses medications outside the trial protocol that may interfere with trial results (e.g., L-carnitine, aromatase inhibitors, hormonal drugs, etc.); (5) Participants are experiencing serious adverse events; (6) Participants who naturally withdraw or are lost to follow-up during observation, including those who respond to treatment but cannot complete the full course, resulting in incomplete clinical data that may affect efficacy and safety evaluation.

#### 2.2.6 Sample size consideration

This trial employed a randomized, double-blind, parallel-group design. Sample size calculations were based on the primary efficacy indicators, estimated as the change in PR from baseline at week 12 of treatment. Based on our preclinical pilot study, acupuncture treatment for asthenozoospermia yielded a mean percentage change of 6.6 ± 4.2, while sham acupuncture treatment showed a mean change of 3.2 ± 3.7. With α = 0.025 (one-tailed) and β = 0.1 (90% power), the critical value was estimated as δ = 5% (1/5 of the mean change). With a 1:1 trial-to-control ratio, each group requires 30 participants. Strict quality control during the trial will limit the loss to follow-up rate to 20%, resulting in a total study sample of 72 participants, and finally 36 in the trial group and 36 in the control group.

#### 2.2.7 Randomization and allocation

The trial will use simple randomization with a 1:1 allocation ratio between the treatment and control groups. Random assignment codes were generated by a statistics professional using SPSS software (Chicago, IL, USA) to create a random number grouping table. During implementation, enrolled participants will be assigned to corresponding groups for intervention and observation based on the sequential numbers in the randomized coding table. Participants will be randomly divided into a treatment group (acupuncture) and a control group (sham acupuncture). A total of 72 participants will be observed. Grouping information will be numbered sequentially and hidden by opaque, and sealed envelopes.

#### 2.2.8 Blinding

In this study, the acupuncture practitioners will be not blinded, while the participants, outcome data collectors, statistical analysts, and outcome assessors will be blinded. Both the acupuncture group and the sham acupuncture group underwent skin penetration, but the location and depth of the acupuncture will be different. Participants will be in separate rooms, positioned supine for acupoint selection, with curtains drawn below the head to obstruct their view of the specific acupuncture locations. Additionally, appointments will be scheduled for the acupuncture sessions to prevent conversations among the participants.

Participants will be informed prior to randomization: You have a 50% chance of being assigned to either the traditional acupuncture group (deeper needle insertion) or the modern acupuncture group (shallow needle insertion). After the final treatment session at Week 12, each participant will complete a questionnaire asking, “Did you receive traditional acupuncture?” with response options of “Yes,” “No,” or “Uncertain.” Ultimately, Bang’s blinding index and James’s blinding index will be used to assess whether participants could distinguish between receiving real acupuncture or sham acupuncture (23, 24).

### 2.3 Intervention

Participants will be randomly assigned to either the acupuncture group or the sham acupuncture group using a random number table, with 36 participants in each group. Acupuncture procedures will be performed by registered traditional Chinese medicine (TCM) physicians from Xiyuan Hospital, China Academy of Chinese Medical Sciences with at least 3 years of clinical experience and trained to treat participants in accordance with the study protocol. Treatments will be conducted in a room maintained at a constant temperature of 26 °C, with participants lying in a supine position. To ensure privacy, each participant will use an individual treatment bed separated by curtains.

#### 2.3.1 Lifestyle intervention

Both the acupuncture group and the sham acupuncture group will receive health education upon enrollment, including suggestions from the researchers to maintain a regular diet, engage in appropriate exercise (Participants will choose a suitable form of exercise and persist with it; Participants will be recommended a combination of aerobic and resistance training, ensuring each session lasts at least 30 min and is performed at least twice a week), maintain good sleep habits, and avoid prolonged sitting (participants will be advised to get up and move for 10–20 min after every 1–2 h of sitting, with no single sitting session exceeding 2 h; they will also be suggested to sleep before 23:00 pm daily, minimizing or avoiding staying up late).

In addition, participants will be suggested not to take or use any Chinese herbal preparations (such as Shengjing Capsules, Qilin Pills, Compound Xuanju Capsules, etc.), health supplements (such as Vitamin E, Coenzyme Q10), drugs (such as L-carnitine, aromatase inhibitors, hormonal drugs, metformin, liraglutide, semaglutide, etc.), or any other health supplements, weight-loss teas that may affect semen quality or body weight from the researcher’s assessment. Additionally, participants will also be advised to avoid hot water sitz baths, moxibustion, acupoint embedding, and physiotherapy during the study period.

#### 2.3.2 Acupuncture group

**FIGURE 2:**
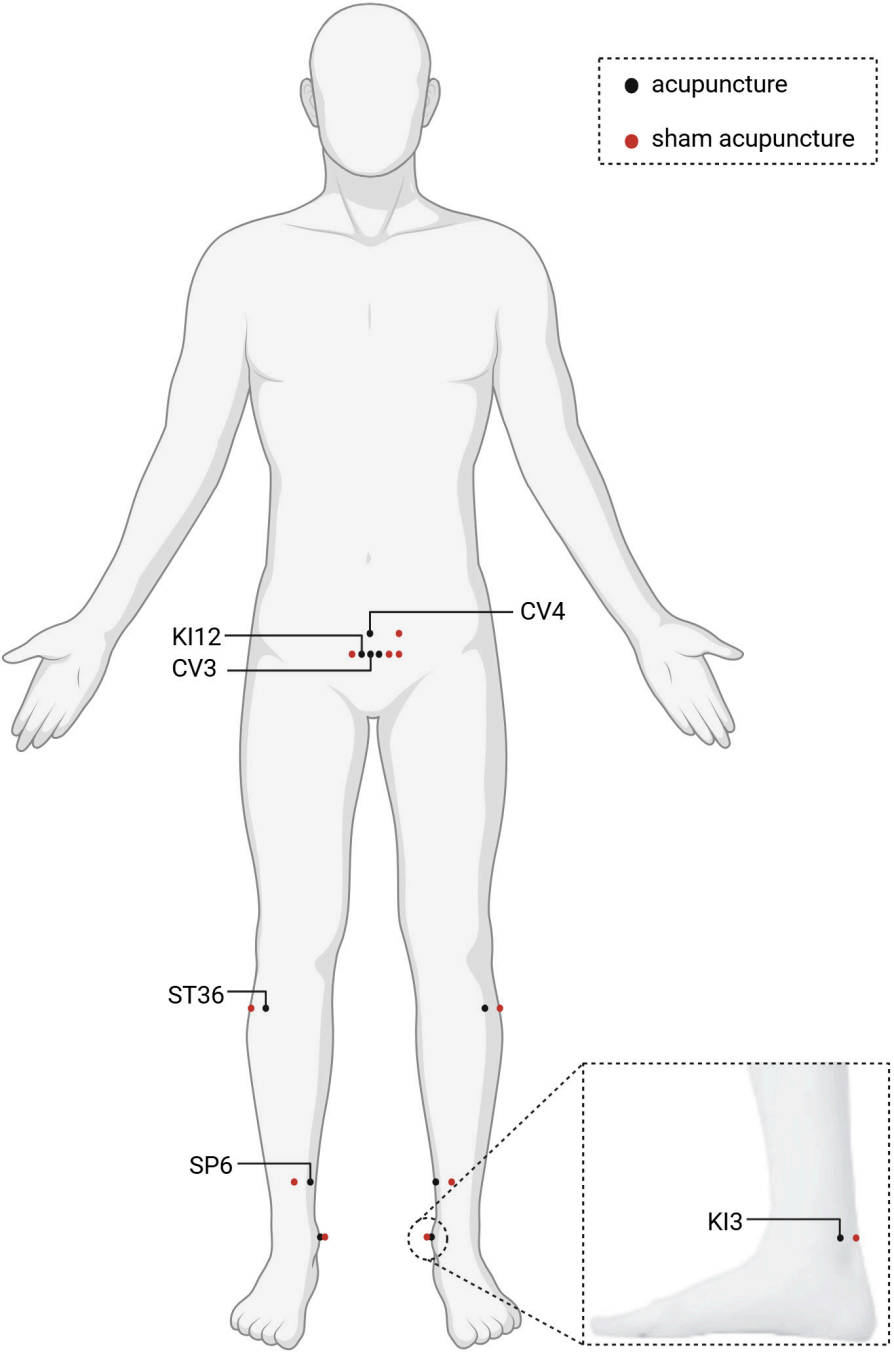
Anatomical locations of acupuncture and sham acupuncture acupoints. Created in BioRender. Wang, H. (2026) https://BioRender.com/nqr7qrs.

We will select bilateral ST36 (Zusanli), bilateral SP6 (Sanyinjiao), CV4 (Guanyuan), CV3 (Zhongji), bilateral KI12 (Dahe), and bilateral KI3 (Taixi) as the treatment acupoints (Figure 2). All acupoints’ location are in Table 2 and Figure 2. They are localized according to the World Health Organization Standard Acupuncture Point Location (24). The 0.3 mm × 40 mm stainless steel milli-needle (Huacheng Brand, Beijing Keyuanda Medical Devices Co., Ltd.) will be used for acupuncture. During the procedure, the acupuncturist should insert the needle gently and slowly until the tip reaches the expected depth. The needle will be manipulated every 10 min with 30 s each time to achieve the deqi sensation. The treatment will span 12 weeks (twice a week, with no less than 2 days between each pair of acupuncture sessions). And each acupuncture session will involve retaining the needle for 30 min.

**TABLE 2:** Intervention in the acupuncture group.

Acupoints	LocationInsert angle	Insert depth
ST36 (Zusanli)	3 cun below ST35 (Dubi), one finger-breadth from the anterior crest of the tibia.90°	2 cun
SP6 (Sanyinjiao)	3 cun directly above the tip of the medial malleolus, on the posterior border of the tibia.90°	1.5 cun
CV4 (Guanyuan)	Front median line of the body, 3 cun below the middle of the navel90°	1.5 cun
CV3 (Zhongji)	Front median line of the body, 4 cun below the middle of the navel90°	1 cun
KI12 (Dahe)	Front median line of the body, 4 cun below the middle of the navel, 0.5 cun next to the anterior median line90°	1 cun
KI3 (Taixi)	Medial side of the foot, depression between the posterior aspect of the medial ankle and the tendon of the heel bone90°	1 cun

#### 2.3.3 Sham acupuncture group

The procedure for sham acupuncture will adhere to the protocol outlined in our previous article (25, 26). Participants will assume a supine posture and undergo standard localized disinfection. Subsequently, the 0.3 mm × 25 mm stainless steel milli-needle (Huacheng Brand, Beijing Keyuanda Medical Devices Co., Ltd.) will be inserted at the acupoint without deqi sensation. Both needle retention time and the overall treatment period will match those applied in the acupuncture group. Specific manipulations will be performed by the acupuncturist: 10 mm lateral to the ST36 (Zusanli), 2–3 mm straight stab; 10 mm anterolateral to the SP6 (Sanyinjiao), 2–3 mm straight stab; 10 mm posterior to the KI3 (Taixi), 2–3 mm straight stab; 5 mm lateral to the KI12 (Dahe), 2–3 mm straight stab; 15 mm lateral to the CV3 (Zhongji), 2–3 mm straight stab; and 15 mm lateral to the CV4 (Guanyuan), 2–3 mm straight stab (Figure 2).

### 2.4 Outcome measures

#### 2.4.1 Primary outcome measure

Progressive sperm motility (PR) refers to the proportion of sperm in semen that can move in a straight line or with a larger radius, which is an important indicator for assessing sperm motility. We will assess the PR at week 12 as the primary outcome.

#### 2.4.2 Secondary outcome measures

The PR data obtained at weeks 4, 8, 18, and 24 will be used as the secondary outcome. The PR plus NP, sperm concentration, semen volume, sperm morphology will be measured by the computer assisted sperm analysis system, and both of these indicators are related to the semen quality. BMI, waist-to-hip ratio, body fat percentage at weeks 4, 8, 12, 18 and 24 compared to that baseline are also considered as secondary outcome measures.

#### 2.4.3 Safety evaluation and adverse events

From previous published clinical studies, potential adverse effects of acupuncture may encompass discomfort, queasiness, emesis, heart palpitations, lightheadedness, cephalalgia, reduced appetite, localized bruising, and infections (27). Furthermore, incidents such as needle fracture, retained needles, or syncopal episodes could arise throughout the therapeutic process. All such unfavorable responses and occurrences will be systematically recorded by the assessing personnel.

### 2.5 Data management and analysis

#### 2.5.1 Data management

The contents of the case report form will be accurately recorded in compliance with the case report form completion guidelines to ensure authenticity and reliability, and subsequently entered into the electronic database. Upon study completion, the paper type of the case report forms will be stored in locked cabinets. And the electronic database will be secured with write-protection to prevent data modification. Eight years after the end of the project, all paper documents need to be shredded, and electronic data need to be deleted.

#### 2.5.2 Data analysis

Statistical analysis will be performed using SPSS (Chicago, IL, USA). For quantitative data (e.g., PR, sperm concentration, semen volume), normality of distribution will be first assessed. For data meeting normality, descriptive statistics will be presented as mean ± standard deviation. Non-parametric rank-sum tests will be used for non-normally distributed data. Count data will be expressed as frequency and percentage [*n* (%)]. For unordered categorical data, chi-square tests will be applied; for ordered categorical data, non-parametric rank-sum tests will be selected. The test level will be set at α = 0.05. *P* < 0.05 indicated statistically significant differences. For repeated measures data, repeated measures analysis of variance (ANOVA) will be performed. If the sphericity test is not satisfied, the results from the Greenhouse–Geisser correction in one-way ANOVA will be used.

### 2.6 Quality control

All relevant personnel will participate will attend specific training about the study objectives, protocol of intervention strategies, and quality control before the study so as to make them understand the protocol and be consistent in the interventions and evaluations during the study process. Before the trial commencement, two TCM practitioners from the selected department will undergo acupuncture technique training. Subsequent interventions will be performed by these two practitioners. The quality control officer will be responsible for establishing the treatment control and quality assurance system for the clinical study. They shall accurately, thoroughly, and meticulously document study case details according to case report form requirements, ensuring content authenticity and reliability before database entry. Upon study completion, paper case report forms will be stored in a locked cabinet. Investigators cannot modify data. All clinical trial results must undergo validation to ensure data reliability and confirm trial conclusions derive from original data. Corresponding data management measures exist throughout clinical trial and data processing phases.

Data collectors will use randomized number codes during data collection. After data collection concludes, the clinical monitor will unblind the primary blind of the randomized case report form, revealing the corresponding A/B codes (without disclosing the specific names of the A/B procedures). Statisticians use A/B codes instead of procedure names during statistical analysis. Following statistical analysis, outcome evaluators assess the efficacy and safety of the A/B procedures. After evaluation, the clinical monitor unmasks the secondary blind layer of the trial case randomization table, revealing whether the A/B intervention was acupuncture or sham acupuncture.

## 3 Discussion

Currently, many studies showed that obesity affects semen quality through multiple mechanisms (28, 29), which also leads to differences in the treatment of obese patients with asthenospermia compared to conventional idiopathic asthenospermia without obesity. Although clinical evidence supported the advantages of bariatric surgery in improving semen quality in obese men, some meta-analyses still indicated limited efficacy of bariatric surgery in this regard (30). Additionally, testosterone replacement therapy and hypoglycemic drugs are associated with poor patient compliance due to many adverse reactions (10). Therefore, exploring safe treatment pathways to meet patients’ fertility needs is still necessary. As an important component of TCM, acupuncture has gradually gained widespread application in metabolic and reproductive diseases in recent years. A lot of meta-analyses support the positive effects of acupuncture in losing weight (31, 32). While male infertility patients can also benefit from acupuncture treatment (33). We have designed this clinical trial focusing the comorbidity, and it would be the first study to confirm the efficacy and safety of acupuncture in the treatment of asthenospermia in obese men.

According to the TCM theory, the main causes of asthenospermia in obese men are kidney deficiency, spleen deficiency, and mostly due to unhealthy lifestyle, such as the high-calorie dietary habit, lack of exercise, and irregular sleep schedule. Patients may benefit from the treatment principle of tonifying the kidney and strengthening the spleen. Therefore, the acupoints with effects of the kidney and spleen based on TCM will be selected. ST36 and SP6 are commonly used to nourishing the spleen and stomach. Also, KI12 and KI3 have been widely used to benefiting the kidney to make it normally governing the male reproductive health. Regarding the use of CV3 and CV4, on the one hand, these two acupoints are located near the male reproductive organs (referred to as the “essence chamber” in TCM), which aligns with the TCM principle that “the location of the acupoint dictates its treatment.” On the other hand, we have used these two acupoints to other andrological diseases based on the “brain-heart-kidney-essence chamber” axis theory in our previously published study and finally achieving clinical efficacy (33, 34). In addition, a systematic review indicated that ST36 and SP6 are the most commonly used acupoints for treating obesity (35); a literature review by Feng et al. (36) also showed that CV4, SP6, KI3, and ST36 are fundamental acupoints for treating male infertility.

Although acupuncture is a safe treatment without any pharmaceutical intervention, to avoid delaying participants’ treatment and potential pregnancy plans, we will exclude participants who could intend to conceive within the next six months. During the intervention, participants will decide whether to use contraceptive measures. Pregnancy of the participant’s partner during the intervention period will not affect the continuation of the study. There are currently no published clinical evidence on acupuncture treatment for obese patients with asthenospermia. Our previously studies have indicated that the frequency of acupuncture for treating andrological diseases is typically 2–3 times per week (37, 38), while the treatment course for male infertility with TCM is usually about three months, which aligns closely with the spermatogenesis cycle (39). Therefore, we have set the treatment regimen to three months, with sessions twice a week. In addition, a defining feature of acupuncture is its sustained therapeutic effect, where patients continue to reap benefits long after the whole treatment ends (40). This established phenomenon is precisely why we have designated a 12-week follow-up period.

Our study also has several limitations. First, TCM emphasizes individualized treatment, meaning that the same disease may be treated with different herbal formulas or acupoints. In our study, all participants will receive the same treatment without individualized adjustments. We will conduct further research to determine appropriate acupoint combinations based on the conclusion that acupuncture has efficacy for obese patients with asthenospermia. Second, the practitioners will not be blinded, and acupuncture techniques (including operational proficiency and needle manipulation methods) could not be completely standardized across different practitioners during the implementation. We will provide training for the practitioners before the trial begins, and implement quality control measures to minimize confounding factors. Third, although we standardized the needle insert depth, the sensation of deqi achieved by the participants may vary due to differences in body size. Finally, Single-center recruitment limits generalizability. We will also conduct a multicenter, large-sample randomized controlled trial to further validate the efficacy of acupuncture in treating asthenospermia in obese men in the future.

## 4 Conclusion

Our random controlled study will represent a significant step forward in acupuncture for obesity-associated reproductive diseases. And this treatment approach can enrich the therapeutic options for obesity-associated asthenospermia, and provide an effective treatment choice for obese men affected by infertility.
